# The effect of salubrinal on the endoplasmic reticulum stress pathway in heat‐stressed spermatogonial cells *in vitro*

**DOI:** 10.1002/2211-5463.70169

**Published:** 2025-11-28

**Authors:** Suna Karadeniz Saygili, Mustafa Oztatlici, Mahmut Kemal Ozbilgin

**Affiliations:** ^1^ Department of Histology and Embriyology, Faculty of Medicine Kutahya Health Sciences University Turkey; ^2^ Department of Histology and Embriyology, Faculty of Medicine Gaziantep Islamic Science and Technology University Turkey; ^3^ Department of Histology and Embriyology, Faculty of Medicine Manisa Celal Bayar University Turkey

**Keywords:** cell culture, endoplasmic reticulum, GC1, GC2, heat stress, salubrinal, spermatogenesis

## Abstract

In this study, we aimed to investigate the effect of salubrinal (SAL) on endoplasmic reticulum stress via an experimental *in vitro* heat stress model (HSM) of spermatogenic cells. In order to achieve this, mouse spermatogonium (GC1) and spermatocyte (GC2) cell lines were used. The IC50 dose of SAL was calculated using an MTT assay. Each cell line was separated into four different groups: control (GC1C, GC2C), SAL‐treated (GC1SAL, GC2SAL), experimental HSM (GC1HSM, GC2HSM), and SAL‐treated HSM (GC1HSMSAL, GC2HSMSAL). Control cells were incubated under standard culture conditions. HSM group cells were incubated at 43 °C for 60 min. In the SAL group, cells were incubated with 20 μm SAL‐containing culture medium for 24 h. Following treatment, all groups were stained with immunofluorescence probes for p‐PERK, ATF6, GRP78, p‐IRE1α, p‐eIF2α, and HSP70 antibodies. Moreover, the mRNA levels of GRP78, PERK, and eIF2α were evaluated via qRT‐PCR. We observed that HSM cells showed cytotoxic effects as all markers showed elevated immunoreactivity levels, which were attributed to ER stress. SAL treatment decreased levels of ER stress. Furthermore, GRP78, PERK, and eIF2α mRNA levels were upregulated in the HSM group and although there was a downregulation following SAL treatment, the difference was not statistically significant. In light of these findings, we concluded that heat stress triggers ER stress in spermatogenic cells, and SAL might affect ER stress markers. Further studies on ER‐related stress mechanisms in spermatogenic cells will be critical in developing therapeutic strategies with advanced molecular analyses in the future.

## Graphical abstract

This study investigates the protective role of salubrinal against heat‐induced endoplasmic reticulum (ER) stress in mouse spermatogenic cells (GC1 and GC2). By modulating the ER stress pathway, salubrinal alleviates cellular stress and supports spermatogenic cell survival, suggesting its potential as a therapeutic candidate for heat‐related infertility.

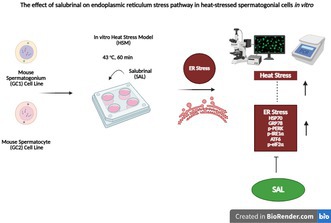

**Abbreviations**
ATF6: activating transcription factor 6
cDNA: complementary deoxyribonucleic acid
CTCF: corrected total cell fluorescence
DAPI: 4′,6‐diamidino‐2‐phenylindole
DMEM: Dulbecco's modified Eagle's medium
DMSO: dimethyl sulfoxide
eIF2α: eukaryotic initiation factor 2 alpha
ER: endoplasmic reticulum
FBS: Fetal bovine serum
GC1: mouse spermatogonium cell line (ATCC® CRL‐2053™)
GC2: mouse spermatocyte cell line (ATCC® CRL‐2196™)
GRP78 (BiP): glucose‐regulated protein 78/binding immunoglobulin protein
HSM: heat stress model
HSP70: heat shock protein 70
IC₅₀: half‐maximal inhibitory concentration
IRE1α: inositol‐requiring enzyme 1 alpha
MTT: 3‐(4,5‐dimethylthiazol‐2‐yl)‐2,5‐diphenyltetrazolium bromide
PBS: phosphate‐buffered saline
PBS‐T: phosphate‐buffered saline with Tween‐20
PERK: protein kinase RNA‐like endoplasmic reticulum kinase
qRT‐PCR: quantitative real‐time polymerase chain reaction
SAL: salubrinal
UPR: unfolded protein response

Spermatogenesis is the process of male germ cell development, which comprises proliferation and differentiation stages called mitosis, meiosis, and spermiogenesis. Endogenous and exogenous biological, physiological, and environmental factors affect this process [1]. In mammals, the spermatogenesis process in the testes occurs at a temperature lower than body temperature. In men, exposure to high temperatures has a serious detrimental effect on fertility and is considered an important risk factor for infertility. Since increased DNA damage and death have been observed in spermatogenic cells subjected to abnormal temperature increases, molecular processes have been elucidated in recent years. For example, transcriptome and experimental investigations have shown that heat exposure activates oxidative stress, endoplasmic reticulum stress, and apoptosis‐related pathways in spermatogenic cells, but many details remain unclear [2, 3].

The endoplasmic reticulum (ER) is a multifunctional organelle essential for cell metabolism, survival, and differentiation. It plays pivotal roles in co‐ and post‐translational protein folding and quality control, the synthesis of membrane and secretory proteins such as hormones and growth factors, calcium storage and signaling, and lipid and sterol biosynthesis [4, 5]. Therefore, proper ER homeostasis is fundamental for cellular adaptation and development. The disruption of ER functions via intracellular or extracellular insults is referred to as ER stress, which leads to the accumulation of unfolded or misfolded proteins and subsequently activates the unfolded protein response (UPR) [6]. While the UPR initially aims to restore proteostasis by upregulating molecular chaperones such as BiP/GRP78, prolonged or severe ER stress triggers apoptotic signaling. High temperatures are a well‐established environmental stressor that disturbs ER homeostasis and initiates UPR activation in mammalian cells, including germ cells [7]. This dual role of the ER in adaptation versus apoptosis highlights its central importance in the context of heat stress‐induced testicular cell injury.

Cells activate a protection mechanism called UPR under prolonged ER stress conditions and struggle to survive under these conditions. In mammals, the UPR signaling pathway is initiated through three transmembrane sensor proteins bound to the ER membrane: IRE1 alpha (inositol‐requiring kinase 1 alpha), PERK (protein kinase‐like ER kinase), and ATF6 (activating transcription factor 6). While the luminal parts of sensor proteins are bound to GRP78, an ER chaperone protein, under normal conditions, dissociation occurs under stress conditions. In a prolonged UPR, ER homeostasis is disrupted, the cell cannot cope with stress, and death mechanisms are activated [8], as previously described in our earlier study [9].

Salubrinal (SAL; *N*‐(3,5‐dibromo‐4‐hydroxybenzylidene)‐amino‐benzohydrazide) is a synthetic phosphatase inhibitor that selectively inhibits eIF2α dephosphorylation through the GADD34/PP1 and CREP/PP1 complexes, thereby extending eIF2α phosphorylation and influencing endoplasmic reticulum stress responses [10, 11, 12]. Previous research indicates that SAL protects against oxidative stress and endoplasmic reticulum stress in neurodegenerative diseases, ischemia–reperfusion injury, metabolic disorders, and cancers [13, 14, 15]. Notwithstanding these defined functions, the capacity of SAL to modulate endoplasmic reticulum stress in germ cells—particularly under thermal stress—remains unexamined. Endoplasmic reticulum stress and eIF2α phosphorylation are recognized as triggers for germ cell apoptosis following thermal exposure. SAL may alleviate heat‐induced endoplasmic reticulum stress in male germ cells, indicating a potential therapeutic approach. The current investigation aimed to establish an *in vitro* heat stress model (HSM) using GC1 and GC2 spermatogenic cell lines and to assess the impact of SAL on ER stress‐related markers, including GRP78, HSP70, ATF6, p‐PERK, p‐eIF2α, and p‐IRE1α. We aimed to determine how SAL influences ER stress signaling in germ cells subjected to hyperthermic conditions by integrating cytotoxicity assays, immunofluorescence, and qRT‐PCR studies. This study establishes a platform for future research on therapeutic approaches to heat‐induced testicular damage.

## Materials and methods

### Cell culture

The mouse spermatogonial GC1 (ATCC® CRL‐2053™ Manassas, VA, USA) and GC2 (ATCC® CRL‐2196™ Manassas, VA, USA) cell lines used in the study were purchased commercially from ATCC (USA). The ready‐to‐use medium was prepared by adding 10% fetal bovine serum (FBS) to Dulbecco's modified Eagle's medium (DMEM). The prepared medium was sterilized by passing it through a 0.2 μm filter before application. The cells were allowed to proliferate by incubating them under sterile conditions in an incubator with 5% CO_2_ at 37 °C.

### Dose modulation of SAL

In this study, 3‐(4,5‐dimethylthiazole‐2‐yl)‐2,5‐diphenyl tetrazolium bromide (MTT) test was used to investigate whether SAL has any toxic effect on cell proliferation and growth. Cells were seeded in 96‐well microplates at 1 × 10^3^ cells per well. Following this, SAL was applied to the cells at ratios ranging from 1 to 100 μm and incubated for 24 h. After treatment, the cells were washed with PBS and left for 1 min, and the PBS was removed. Then, 100 μL of fresh culture medium and 10 μL of MTT stock solution were added to each well. The wells were wrapped in aluminum foil and kept in the incubator at 370 °C for 4 h. After incubation, the solution was removed, and the culture medium containing MTT was pipetted. Then, 100 μL DMSO was added to the cells and kept at room temperature for 10 min. Absorbance at 570 nm was measured in a microplate reader spectrophotometer. The whole experimental protocol was repeated 3 times.

### Creation of *in vitro* heat stress model

The *in vitro* heat stress model (HSM) was applied by exposing GC1 and GC2 cells to 43 °C for 60 min in a humidified incubator. This protocol was selected based on preliminary experiments and previously published studies that indicated this condition is widely used to induce heat stress in germ cell culture models [16]. At the end of the exposure period, the incubator temperature was adjusted back to 37 °C. For each cell line, four experimental groups were created: GC1 cells—GC1C (control, cultured under standard conditions), GC1SAL (treated with 20 μm Salubrinal without heat stress), GC1HSM (exposed to heat stress at 43 °C for 60 min), and GC1HSMSAL (exposed to heat stress at 43 °C for 60 min followed by treatment with 20 μm SAL); GC2 cells—GC2C (control, cultured under standard conditions), GC2SAL (treated with 20 μm Salubrinal without heat stress), GC2HSM (exposed to heat stress at 43 °C for 60 min), and GC2HSMSAL (exposed to heat stress at 43 °C for 60 min followed by treatment with 20 μm SAL). The cells were then incubated for 24 h. After 24 h, the cells were fixed in 95% ethanol for 15 min for analysis.

### Immunofluorescence staining

GC1 and GC2 cells were seeded at a density of 1 × 10^5^ cells per well in 6‐well plates and allowed to adhere overnight before experimental treatments. After immunofluorescence staining treatments, slides were incubated in a humidified chamber. The cell smear on the slide surface was circled with a hydrophobic barrier pen. After rinsing briefly with distilled water, endogenous peroxidase activity was blocked with 3% hydrogen peroxide treatment for 5 min. The samples were washed for 3 min with PBS containing 0.1% Tween‐20 (PBS‐T). After that, cells were incubated with blocking serum for 30 min. HSP70 (Biorbyt, orb157591, 1 : 200), p‐eIF2α (Bioss, BS‐4842, 1 : 200), p‐PERK (Bioss, bs‐3330R, 1 : 200), p‐IRE1α (Biorbyt, orb157704, 1 : 200), ATF6 (Bioss, bs‐1634R, 1 : 200), and GRP78 (Bioss, bs‐1219R, 1 : 200) rabbit polyclonal primary antibodies were incubated overnight for approximately 16 h at 4 °C in a humidified chamber. Cells were washed with PBS‐T for 3 min and incubated with 1 : 500 anti‐rabbit IgG Alexa Fluor 488 Conjugate (Thermo, CST #4412, Danvers, MA, USA) and anti‐mouse IgG Alexa Fluor 555 Conjugate (Thermo, CST #4409, Danvers, MA, USA) secondary antibodies for 1 h in a dark environment at room temperature. It was then closed with 4',6‐diamidino‐2‐phenylindole (DAPI) and mounting medium prepared in a 1 : 1 ratio. Fluorescence images were obtained using a Zeiss Axio Observer fluorescence microscope (Carl Zeiss Microscopy GmbH, Jena, Germany) equipped with a high‐resolution digital camera. Image acquisition and processing were performed using zen imaging software (Zeiss, version 3.4), and identical exposure times and gain settings were applied across all experimental groups. Images were taken in tiff format in 10 different areas at random 20× magnification from each group. After the images were transferred to image j Analysis Software (v1.48, National Institutes of Health, Bethesda, Maryland, USA), fluorescence measurements were made. After the measurement, the CTCF (Corrected Total Cell Fluorescence) values were calculated using the formula CTCF = Whole density − (Selected Area × Average of Background Fluorescence Reading) [17].

### qRT analysis

GC1 and GC2 cells were seeded at a density of 1 × 10^5^ cells per well in 6‐well plates and allowed to adhere overnight before experimental treatments. For the qRT‐PCR analysis, at the end of the experimental treatments, the cells in all groups were washed with 500 μL PBS before RNA isolation. After washing, they were centrifuged for 10 min at 300 ***g*** in 500 μL PBS. After centrifugation, the supernatant was removed. RNA isolation was performed according to the protocol recommended by the manufacturer (Ambion, Purelink RNA Mini Kit, Floor no. 12183018A, Waltham, Ma,USA). Qualitative and quantitative analyses of the obtained RNA samples were performed via spectrophotometric measurement with the Maestro NanoDrop device. The RNA obtained from the cells was translated into cDNA without waiting. High‐Capacity cDNA Reverse Transcription Kit for cDNA synthesis (Applied Biosystems, Cat. No: 4368814, Waltham, MA, USA) was used. We aimed to measure the mRNA expression amounts of PERK, GRP78, and EIF2A produced in cells after cDNA synthesis. The primary probes used in qRT‐PCR were received ready‐made from the manufacturer (SentebioLab, Ankara, Turkey). Suitable primers for PERK, GRP78, and eIF2a mRNA sequences for the qRT‐PCR study review were designed using the web‐based software program primer3 (http://bioinfo.ut.ee/primer3‐0.4.0/). The mRNA sequences used in the study are shown in Table 1. PowerUp Syber Green Kit was used for qRT‐PCR gene analysis, and all analyses were repeated 3 times under the same and sterile conditions, respectively, to minimize experimental errors. Data were taken at the end of the analysis, and each sample was normalized according to the expression of the reference gene. The expression differences between the samples were evaluated according to the calculation of $2^{-\Delta \Delta C_{t}}$. Dec.

**Table 1:** The forward (F) and reverse (R) primer sequences employed for the quantitative real‐time PCR (qRT‐PCR) analysis of PERK, eIF2α, and GRP78 genes. The base pair column indicates the nucleotide length of each primer.

Gene	Primer sequence (5′–3′)	Base pairs
PERK	F: GGTATTTCAACGCCTGGCTG R: GGCCAGTCTGTGCTTTCGTC	20
eIF2α	F: TTCGACCTCCCAAAAGCAGT R: TTGTAGGTTAGGCGTCCCAG	20
GRP78	F: GGCGTGAGGTAGAAAAGG R: ATGGTAGAGCGGAACAGG	18

### Statistical analysis

All experiments were performed in three independent biological replicates (*n* = 3). For immunofluorescence analyses, 10 randomly selected microscopic fields were evaluated per group, and for qRT‐PCR analyses, each sample was analyzed in technical triplicate. Data distribution was assessed using the Shapiro–Wilk normality test. For normally distributed data, one‐way ANOVA followed by Sidak's *post hoc* test was applied. For non‐normally distributed data, the Kruskal–Wallis test with Dunn's *post hoc* correction was used. A *P*‐value of ≤ 0.05 was considered statistically significant. The numerical data obtained were evaluated using graphpad prism 8.3.0 licensed statistical analysis program (GraphPad SoftwareInc., San Diego, CA, USA).

## Results

### SAL cell line MTT results

This experiment examined the cytotoxic effects of Salubrinal on the GC1 and GC2 cell lines, as well as their IC50 values. The results established the experimental foundation for determining the appropriate Salubrinal concentration for further analyses. In experiments conducted under *in vitro* conditions, the results of MTT showed increased dose applications of SAL (1, 5, 10, 25, 50, 75, 100). The effects of μm on viability in the GC1 and GC2 cell lines were evaluated. As a result of the MTT analysis, the IC50 values of SAL in the GC1 and GC2 cell lines were 46.5564 and 42.691 μm, respectively. The percentage of inhibition increased with increased concentration levels.

### SAL cell line application findings

**Fig. 1:**
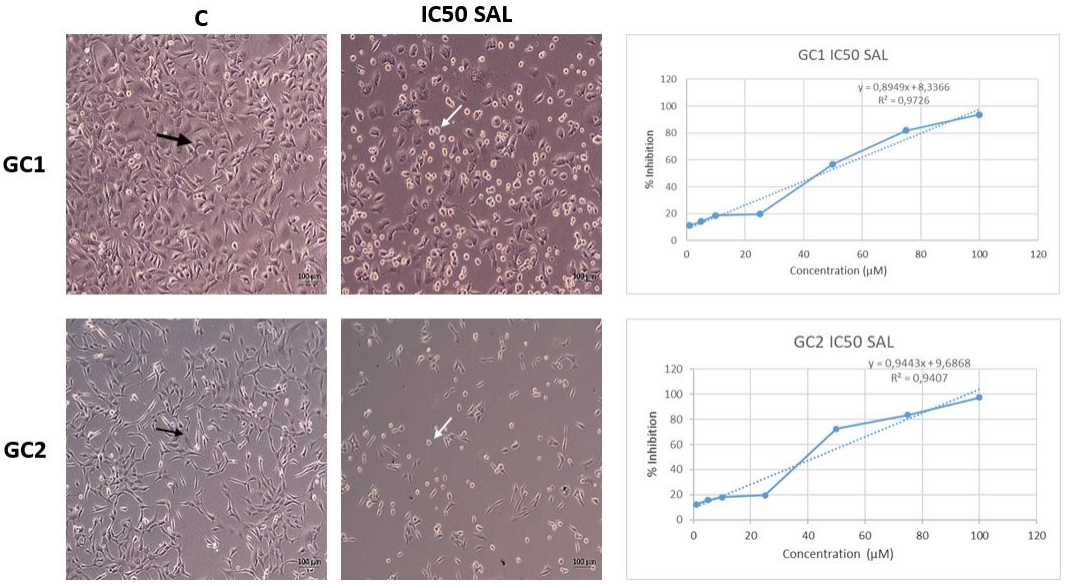
Determination of the IC50 values (μm) for GC1 and GC2 cell lines following 24 h SAL exposure. Representative phase‐contrast images demonstrate the typical epithelioid morphology of cells maintained under standard culture conditions and the reduced viability observed at the IC50 concentration of SAL. The cell images on the left side represent control (C) cells, while the images on the right side represent cells treated with IC50 concentrations of SAL (IC50 SAL). Black arrow: Living cell. White arrow: Dead cell. Scale bar: 100 μm.

This investigation aimed to assess the morphological responses and overall tolerance of GC1 and GC2 cells to varying doses of Salubrinal to ascertain its cellular effects under standard culture conditions. The GC1 and GC2 cell lines were found to exhibit epithelioid characteristics when no application was made under normal conditions (Fig. 1). The application of 20 μm SAL, selected as the experimental application dose, had minimal inhibition on the cells. It was observed that the cells retained their epithelioid characters during application. When administered at a dose of IC50, it showed a serious degree of cytotoxicity and cytopathic effect on the cells. Morphologically, the cells became round and shrank in volume (Fig. 1).

### Heat stress application findings

**Fig. 2:**
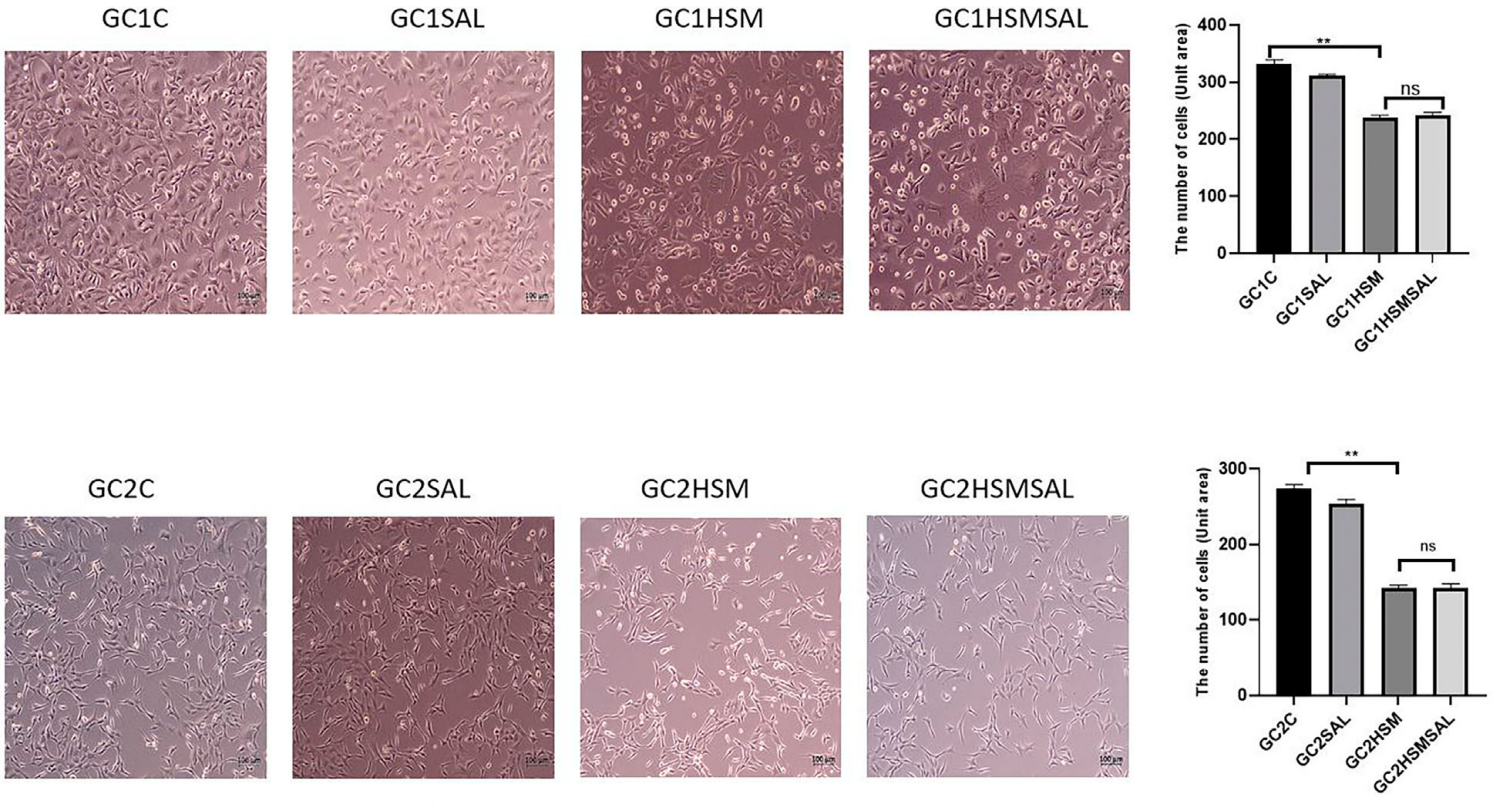
Inverted microscope images of GC1 and GC2 experimental groups. Quantitative analysis shows a decrease in viable cell numbers in the HSM‐applied groups compared to controls. Data are expressed as mean ± SD (*n* = 3, 10 randomly selected fields per group). Statistical analysis: one‐way ANOVA, followed by Sidak's *post hoc* test. (**P < 0.05; statistical analysis was performed using graphpad Software). Scale bar: 100 μm.

This experiment aimed to examine the morphological impacts of heat stress on GC1 and GC2 cells and assess the potential influence of SAL treatment on cellular responses under stress conditions. In the GC1C and GC2 groups without heat treatment, the cells retained their morphological characteristics and were confluent. In the GC1SAL and GC2SAL groups, it was observed that the cells retained their morphological characteristics. In the GC1HSM and GC2HSM groups, there was a decrease in the number of cells and a cytopathic effect on the cells. In the GC1HSMSAL and GC2HSMSAL groups, morphological changes, cytopathic activity, and numerical decreases in cells were observed (Fig. 2).

### Immunofluorescence examination findings

**Fig. 3:**
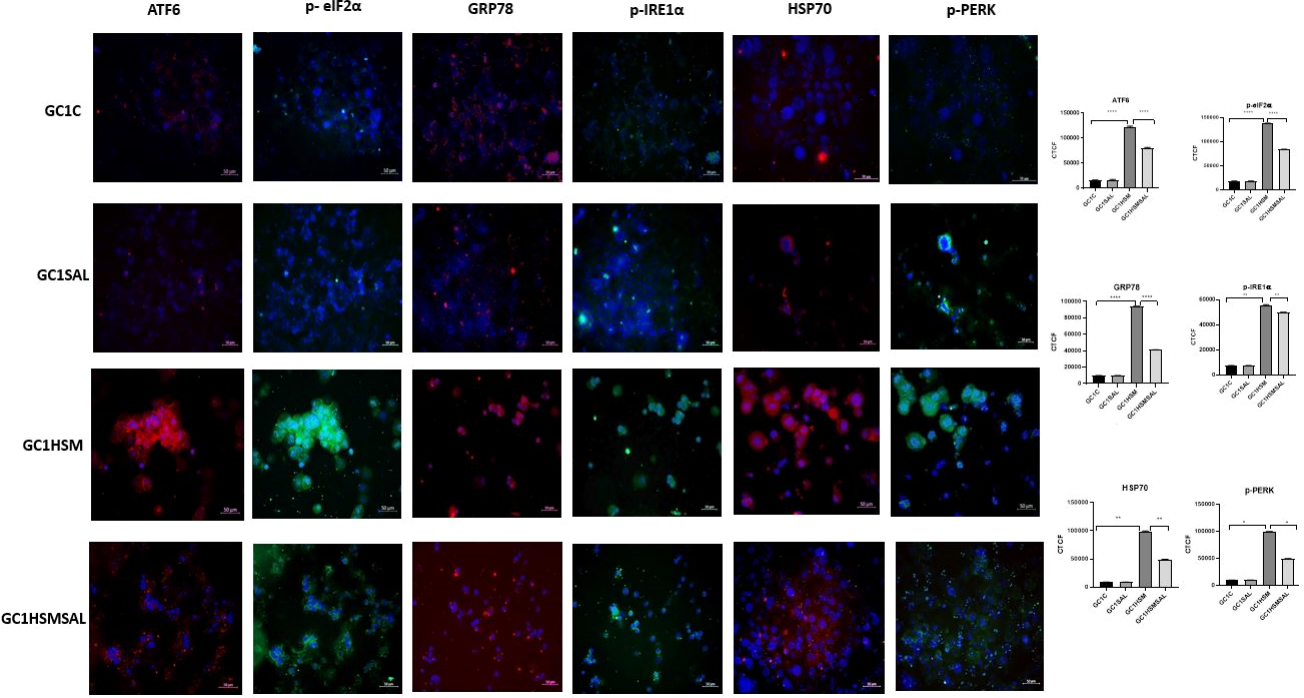
Representative fluorescent images (each image is an overlay with DAPI nuclear staining) and quantitative CTCF values for HSP70, GRP78, p‐IRE1α, p‐PERK, p‐eIF2α, and ATF6 in GC1 cells across all groups (C, SAL, HSM, HSMSAL). Composite images were generated by merging individual fluorescence channels. Data are expressed as mean ± SD (*n* = 3, 10 fields/group). Statistical analysis: one‐way ANOVA, followed by Sidak's *post hoc* test. (*P < 0.05, **P < 0.01, ***P < 0.001, ****P < 0.0001; statistical analysis was performed using graphpad Software). Scale bar: 50 μm.

The present study aimed to evaluate the impact of heat stress and Salubrinal treatment on the expression of ER stress‐related proteins in GC1 and GC2 cells by immunofluorescence labeling. After HSP70, GRP78, p‐IRE1α, p‐PERK, p‐eIF2α, and ATF6 immunofluorescence staining, CTCF values were calculated in 10 different fields at 20× magnification from each group. After statistical evaluation, no significant difference was found between the GC1C and GC1SAL groups (*P* ≥ 0.05). CTCF values were significantly increased in the GC1‐HSM group compared to the GC1‐C group (*P* ≤ 0.05). A statistically significant decrease in CTCF values was found in the GC1HSMSAL group that received SAL compared to the GC1HSM group (*P* ≤ 0.05). Statistical data evaluating the differences between the groups were summarized graphically (Fig. 3).

**Fig. 4:**
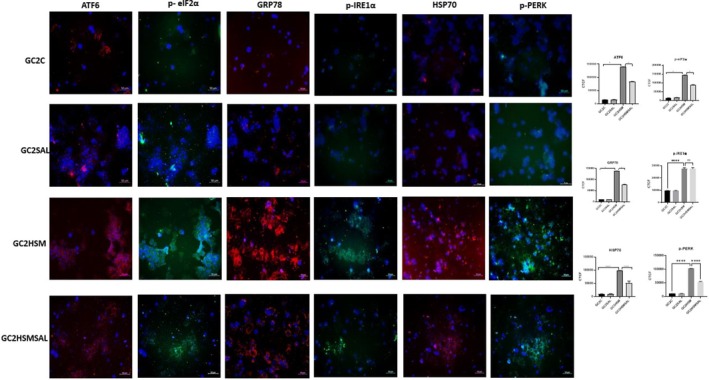
Representative fluorescent images (each image is an overlay with DAPI nuclear staining) and quantitative CTCF values for HSP70, GRP78, p‐IRE1α, p‐PERK, p‐eIF2α, and ATF6 in GC2 cells across all groups (C, SAL, HSM, HSMSAL). Composite images were generated by merging individual fluorescence channels. Data are expressed as mean ± SD (*n* = 3, 10 fields per group). Statistical analysis: one‐way ANOVA, followed by Sidak's *post hoc* test. (*P < 0.05, **P < 0.01, ***P < 0.001, ****P < 0.0001; statistical analysis was performed using graphpad Software). Scale bar: 50 μm.

After HSP70, GRP78, p‐IRE1α, p‐PERK, p‐eIF2α, and ATF6 immunofluorescence staining, CTCF values were calculated in 10 different fields at 20× magnification from each group. After statistical evaluation, no significant difference was found between the GC2C and GC2SAL groups (*P* ≥ 0.05). CTCF values were found to be significantly increased in the GC2HSM group compared to the GC2C group (*P* ≤ 0.05). A statistically significant decrease in CTCF values was found in the GC2HSMSAL group that received SAL compared to the GC2HSM group (*P* ≤ 0.05). Statistical data evaluating the differences between the groups were summarized graphically (Fig. 4).

### qRT‐PCR findings

**Fig. 5:**
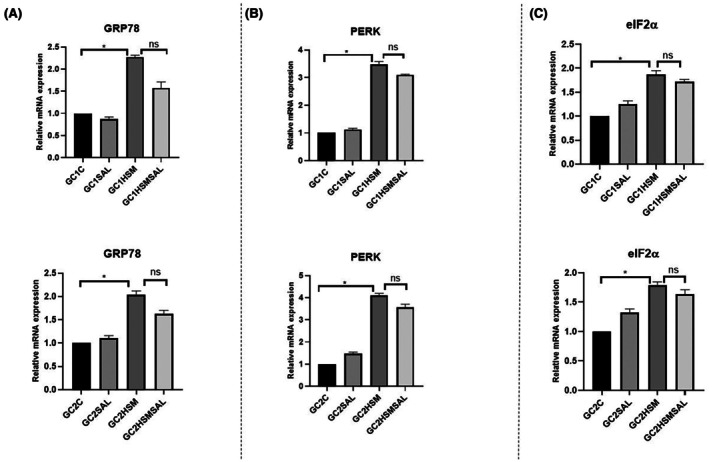
Relative mRNA expression levels of GRP78 (A), PERK (B), and eIF2α (C) in GC1 and GC2 cells. Gene expression was quantified by qRT‐PCR and analyzed using the $2^{-\Delta \Delta C_{t}}$ method. The control groups (GC1C and GC2C) were designated as calibrators and normalized to a fixed value of 1.0 by definition. Data are presented as mean ± SD from three independent biological replicates, each analyzed in technical triplicates. Individual data points are shown for all groups. Statistical evaluation was performed using the Kruskal–Wallis test, followed by Dunn's *post hoc* test (*P < 0.05; statistical analysis was performed using graphpad Software).

The purpose of this experiment was to evaluate the effects of SAL treatment on the mRNA expression levels of ER stress‐related genes (GRP78, PERK, and eIF2α) in GC1 and GC2 cells under heat stress conditions, using quantitative real‐time PCR analysis. qRT‐PCR analysis was performed to determine whether heat stress and SAL application were effective on GRP78 mRNA expression levels. For the GC1 cell line, it was observed that the expression of the GRP78 gene increased by approximately 2.5 times in the GC1HSM compared to the GC1C group. It was determined that the decrease in the relative mRNA expression of GRP78 in the GC1HSMSAL group did not reach statistical significance compared to the GC1HSM group (*P* ≥ 0.05). For the GC2 cell line, it was observed that the expression of the GRP78 gene increased 2‐fold in the GC2HSM compared to the GC2C group. The decrease in the relative mRNA expression of GRP78 in the GC2HSMSAL group exhibited lower values without statistical significance compared to the GC2HSM group (*P* ≥ 0.05) (Fig. 5A).

qRT‐PCR analysis was performed to determine whether heat stress and SAL application were effective in modulating PERK mRNA expression levels. For the GC1 cell line, it was observed that the expression of the PERK gene increased approximately fourfold in the GC1HSM compared to the GC1C group. It was determined that the decrease in the relative mRNA expression of PERK in the GC1HSMSAL group was not statistically significant compared to the GC1HSM group (*P* ≥ 0.05). For the GC2 cell line, it was observed that the expression of the PERK gene increased approximately fourfold in the GC2HSM compared to the GC2C group. It was determined that there was a decrease in the relative mRNA expression of PERK in the GC2HSMSAL group, although this did not reach statistical significance compared to the GC2HSM group (*P* ≥ 0.05) (Fig. 5B).

qRT‐PCR analysis was performed to determine whether heat stress and SAL application were effective in modulating eIF2α mRNA expression levels. For the GC1 cell line, it was observed that the expression of the eIF2α gene increased approximately twofold in the GC1HSM compared to the GC1C group. It was determined that the decrease in the relative mRNA expression of eIF2α in the GC1HSMSAL group did not reach statistical significance compared to the GC1HSM group (*P* ≥ 0.05). For the GC2 cell line, it was observed that the expression of the eIF2α gene increased approximately twofold in the GC2HSM compared to the GC2C group. The decrease in the relative mRNA expression of eIF2α in the GC2HSMSAL group exhibited lower expression, although not statistically significant, compared to the GC2HSM group (*P* ≥ 0.05) (Fig. 5C).

## Discussion

Spermatogenesis requires a temperature moderately lower than core body temperature, and thermal stress is a known important factor contributing to male infertility by causing DNA damage and apoptosis in germ cells [1]. Recent investigations indicate that testicular heat changes spermatogenic gene expression and adversely affects reproductive outcomes [18, 19]. A primary mechanism driving this process is endoplasmic reticulum (ER) stress, marked by protein misfolding and the activation of the unfolded protein response (UPR) via the PERK, IRE1α, and ATF6 pathways [20]. Although brief stimulation of the unfolded protein response (UPR) is beneficial, chronic endoplasmic reticulum (ER) stress induces apoptosis and impairs testicular function [21]. Salubrinal (SAL), an inhibitor of eIF2α dephosphorylation, has demonstrated efficacy in mitigating ER stress and enhancing cellular resilience across many paradigms [22]. This study aimed to investigate the effect of SAL on ER stress sensor proteins in spermatogenic cells formed via HSM *in vitro*.

This study identified the IC50 values of salubrinal (SAL) as 46.5 and 42.7 μm for GC1 and GC2 cells, respectively. Consequently, a dose of 20 μm was chosen for further studies, as it exhibited negligible cytotoxic or cytopathic effects. Our data align with prior studies indicating that SAL displays concentration‐dependent effects, where elevated dosages (> 40 μm) cause cytotoxicity, while lower doses (5–20 μm) have minimal to no detrimental effect on cell viability [23, 24]. Likewise, research on endothelium and other mammalian cell types substantiates that SAL preserves a favorable safety profile at concentrations of ≤ 20 μm [22, 25]. We must consider that IC₅₀ values may fluctuate based on the cell line and experimental parameters [26]. In light of these results, the 20 μm dose value selected as the administration dose was evaluated as an indicator that may vary depending on the cell type and application conditions.

In our model, exposing GC1 and GC2 cells to heat stress (43 °C, 60 min) substantially decreased cell viability, signifying the activation of apoptotic pathways. The finding agrees with the fundamental requirement of germ cells for an appropriate thermal environment that supports biological functions [27]. Multiple *in vitro* investigations have demonstrated the adverse effects of heat stress on cellular viability, showing increased apoptosis in Sertoli cells, germ cells, and somatic cell lines [28, 29]. Our findings align with previous research and indicate that the numerical reduction in GC1 and GC2 cells observed is predominantly attributable to apoptosis induced by heat stress. Recent studies demonstrate that heat stress‐induced cell death is intricately associated with endoplasmic reticulum stress and the activation of the unfolded protein response, thereby reinforcing the molecular interpretation of our findings [4, 7]. In this study, it was also thought that the decrease in the number of cells in GC1 and GC2 cell lines was caused by the application of the heat stress model.

Heat stress markedly elevated HSP70 expression in GC1 and GC2 cells, validating its function as a biomarker for germ cell stress and apoptosis [30, 31]. SAL therapy decreased HSP70 levels, aligning with its ability to mitigate ER stress and protein accumulation [10, 22]. The data indicate that SAL provides cytoprotection, in part by inhibiting HSP70‐mediated stress responses.

Our study demonstrated significantly upregulated GRP78 expression in GC1 and GC2 cells following heat stress, as validated by immunofluorescence and qRT‐PCR, thereby confirming its status as a hallmark of ER stress. GRP78 (BiP/HSPA5) is a pivotal endoplasmic reticulum chaperone that governs protein folding, endoplasmic reticulum‐associated degradation, calcium homeostasis, and the activation of unfolded protein response sensors [20]. Elevated GRP78 levels during hyperthermia have been documented in testicular and somatic cell models [32, 33]. Salubrinal therapy markedly diminished GRP78 expression in both cell lines, signifying reduced ER stress, corresponding with research on oocytes and neural models indicating that SAL reduces GRP78 levels [10, 34, 35]. Nonetheless, context‐dependent effects have been noted, as SAL elevated GRP78 levels in vascular smooth muscle cells [36]. The data indicate that, whereas GRP78 activation signifies ER stress in spermatogenic cells, the impact of SAL on GRP78 may differ based on the cellular environment.

Heat stress significantly increased ATF6 expression in GC1 and GC2 cells, highlighting the activation of the ATF6 pathway of the UPR in germ cell endoplasmic reticulum stress [37]. In contrast, salubrinal therapy decreased ATF6 levels, consistent with its established function in alleviating ER stress signaling [7]. The data indicate that ATF6 modulation may be a crucial mechanism responsible for salubrinal's protective actions against heat‐induced endoplasmic reticulum stress in spermatogenic cells.

Heat stress markedly increased p‐IRE1α expression in GC1 and GC2 cells, thereby validating the activation of the IRE1α pathway of the UPR. IRE1α, a conserved endoplasmic reticulum stress sensor, experiences dimerization and phosphorylation following its dissociation from GRP78, which initiates downstream stress signaling [20]. Prior research has also reported an increase in p‐IRE1α in testicular hyperthermia models [28, 32]. Salubrinal notably decreased p‐IRE1α levels in GC1 cells, but not in GC2 cells, indicating cell type‐specific effects. Context‐dependent modulation of p‐IRE1α by SAL has been noted in oocyte complexes, fibroblasts, and cardiac stress models [24, 38, 39]. The findings suggest that although IRE1α is actively involved in germ cell ER stress, the effects of SAL application on cells on p‐IRE1α levels may be specific.

Our investigation revealed that heat stress markedly elevated p‐PERK expression and PERK mRNA levels in GC1 and GC2 cells, thereby demonstrating the activation of the PERK pathway inside the UPR. PERK is a type I endoplasmic reticulum transmembrane kinase that phosphorylates eIF2α, hence diminishing global protein production and facilitating stress‐adaptive gene expression [20]. In alignment with other studies on germ cells, cardiomyocytes, and epithelial cell models, hyperthermia and oxidative stress significantly stimulate PERK signaling [33, 40]. Salubrinal administration markedly diminished p‐PERK levels in both cell lines, corroborating its function as an ER stress modulator. Comparable PERK inhibition via SAL has been recorded in fibroblasts and hepatic damage models [10, 24]. These data suggest that SAL might protect spermatogenic cells from heat‐induced ER stress, in part by inhibiting PERK.

Heat stress significantly increased p‐eIF2α expression in GC1 and GC2 cells, signifying the activation of the PERK–eIF2α pathway of the UPR. eIF2α phosphorylation diminishes global translation and is typically considered a cytoprotective strategy that restricts the protein load on the endoplasmic reticulum [20, 41]. Comparable elevations of p‐eIF2α after application of hyperthermic or oxidative stress have been documented in germ cells and epithelial models [32, 33]. In our investigation, salubrinal diminished p‐eIF2α levels in both cell lines, aligning with its function as an inhibitor of eIF2α dephosphorylation. Prior research has demonstrated context‐dependent results, with SAL either augmenting or diminishing p‐eIF2α based on cell type and stressor [42, 43]. Consequently, although our observations indicate that SAL has an ER stress‐suppressive impact in spermatogenic cells, the dual function of eIF2α phosphorylation in pro‐apoptotic versus anti‐apoptotic signaling requires further elucidation.

Our findings indicate that salubrinal (SAL) mitigates heat stress‐induced ER stress in GC1 and GC2 cells by inhibiting eIF2α dephosphorylation and modulating the PERK–eIF2α, ATF6, and IRE1α pathways. Given the pivotal role of ER stress in germ cell apoptosis and infertility, these results suggest that pharmacological targeting of the SAL‐regulated pathways may offer therapeutic potential in conditions associated with testicular hyperthermia, oxidative stress, and reproductive dysfunction. Preclinical studies in other models have demonstrated SAL's cytoprotective effects in ischemia–reperfusion injury, metabolic disorders, and neurodegenerative diseases through the same ER stress‐regulatory mechanisms [10, 11, 24]. Integrating our *in vitro* evidence with these findings highlights the possibility of developing SAL or related ER stress modulators as pharmacological interventions to preserve spermatogenesis and male fertility under stress conditions.

This research includes several limitations. The investigations were conducted exclusively *in vitro* with GC1 and GC2 spermatogonial cell lines, potentially failing to represent the physiological complexities of the testis. Secondly, only a restricted array of ER stress markers was assessed, and additional components of the unfolded protein response (e.g., downstream targets of ATF6 and IRE1α) were not comprehensively investigated.

## Conclusions

This study presented a heat stress model in spermatogenic GC1 and GC2 cells to examine endoplasmic reticulum stress responses and the potential protective effects of salubrinal (SAL). The IC₅₀ values of SAL were determined to be 46.5 and 42.6 μm for GC1 and GC2, respectively, with a nontoxic concentration of 20 μm used for the following tests. Heat exposure markedly decreased cell viability and triggered the activation of ER stress indicators, including p‐PERK, p‐eIF2α, ATF6, GRP78, HSP70, and p‐IRE1α. Conversely, SAL therapy diminished these responses, as validated via immunofluorescence and qRT‐PCR analysis. Our data collectively demonstrate that heat stress significantly generates endoplasmic reticulum stress in spermatogenic cells, while SAL successfully alleviates these effects, underscoring its potential as a cytoprotective drug against heat‐induced testicular injury.

Future investigations should try to confirm these results *in vivo* utilizing animal models of heat‐induced testicular injury. Further investigations evaluating more varieties of ER stress and apoptotic indicators might yield enhanced understanding of the molecular mechanisms underlying SAL‐mediated protection. Moreover, extensive investigations are necessary to assess whether the pharmacological regulation of ER stress can result in sustained spermatogenesis and fertility results.

Salubrinal's modulatory effects on ER stress markers were the focus of this work, although its possible protective efficacy under heat stress conditions would be a desirable future research topic. Salubrinal's ability to modulate ER stress and protect spermatogonial cells *in vivo* may reveal its therapeutic potential for male infertility.

## Conflict of interest

The authors declare no conflict of interest.

## Author contributions

Conceptualization, SKS; methodology, SKS and MÖ; investigation, SKS and MKÖ; data curation, SKS; writing – original draft preparation, SKS; writing – review and editing, SKS; supervision, SKS. All authors have read and agreed to the published version of the manuscript.

## Data Availability

All data generated or analyzed during this study is included in this published article.
